# The relationship between the severity of perimenstrual symptoms and a regular exercise habit in Japanese young women: a cross-sectional online survey

**DOI:** 10.1186/s12905-022-01720-2

**Published:** 2022-05-28

**Authors:** Rami Mizuta, Noriaki Maeda, Makoto Komiya, Yuta Suzuki, Tsubasa Tashiro, Kazuki Kaneda, Shogo Tsutsumi, Honoka Ishihara, Sayo Kuroda, Yukio Urabe

**Affiliations:** 1 Graduate School of Biomedical and Health Sciences, Hiroshima University, 1-2-3 Kasumi, Minami-ku, Hiroshima, 734-8553 Japan; 2 Department of Rehabilitation, Matterhorn Rehabilitation Hospital, Hiroshima, 737-0046 Japan

**Keywords:** Perimenstrual symptoms, Menstruation, Dysmenorrhea, Premenstrual syndrome, Japanese, Young women, Exercise habit, Lifestyle, Physical activity, Non-pharmacological coping strategy

## Abstract

**Background:**

Experiencing perimenstrual symptoms is a major health concern for young women. Women in the reproductive age experience menstruation about once a month, and many experience perimenstrual symptoms. Drug therapies such as painkillers (commonly used) and hormonal medications help to achieve symptomatic relief from perimenstrual symptoms. However, many women are concerned about tolerance and compliance of the drugs used to treat perimenstrual symptoms and lack awareness on how to cope with perimenstrual symptoms. If the association between exercise habits and the severity of symptoms is emphasized in young women, methods for coping with perimenstrual symptoms can be established, not relying only on pharmaceuticals. The objective of this study was to determine the differences in the severity of perimenstrual symptoms in young Japanese women with and without a regular exercise habit.

**Methods:**

A cross-sectional study using an online survey was sent among 500 Japanese women aged 18–25 years. The severity of perimenstrual symptoms was assessed using the Menstrual Distress Questionnaire (MDQ). The differences in MDQ scores between those with and without exercise habits (exercise for more than 30 min for 2 days a week) were compared using the chi-square test and Mann–Whitney U test. The logistic regression analysis detected subscales of the premenstrual and menstrual symptoms affected by an exercise habit.

**Results:**

282 (56.4%) young Japanese women were answered this survey. Respondents were divided into the exercise group (n = 157) and the non-exercise group (n = 125). The exercise group had significantly low premenstrual and menstrual MDQ scores. The results of the logistic regression analysis stated that the subscale related to negative emotion before menstruation was associated with an exercise habit. In the analysis performed during menstruation, a statistically significant association was detected between an exercise habit and a behavioral change such as avoiding interaction with others.

**Conclusions:**

This study suggested that exercise habits may reduce the severity of perimenstrual symptoms and could help to develop a non-pharmacological coping strategy. In addition, this study provides useful information for young women who want to prevent perimenstrual symptoms but do not have an exercise habit. Further, it may encourage young women to start exercising.

**Supplementary Information:**

The online version contains supplementary material available at 10.1186/s12905-022-01720-2.

## Background

Perimenstrual symptoms caused by cyclical fluctuations of female hormones are a major health problem for women [1]. Women of reproductive age experience menstruation about once a month, and about 80% of women suffer from perimenstrual symptoms, such as abdominal pain, depression, and avoidance of social activities, in each cycle [2]. Previous studies have shown that perimenstrual symptoms were associated with poor academic performance, work efficiency, and quality of life [3–5]. In addition, the socioeconomic loss in Japan was estimated to be about 683 billion Japanese yen per year after calculating drug therapy for perimenstrual symptoms and labor loss [6]. Therefore, perimenstrual symptoms are a serious problem not only for women but also for society.

Many women lack awareness of how to cope with perimenstrual symptoms. According to a study related to self-care of perimenstrual symptoms, 43.6% of Japanese women mentioned that they would just lie down to relieve their symptoms [7]. In addition, painkillers are commonly used for symptomatic relief, and hormonal medication also reduces perimenstrual symptoms [6]. On the other hand, some people are concerned about drug tolerance and compliance [8]. Thus, it is necessary to establish non-pharmacological coping strategies that are easy to implement. Perimenstrual symptoms are greatly influenced by lifestyle, including diet and alcohol consumption [9, 10]. Exercise has been shown to reduce chronic pain and swelling [11, 12] and has been suggested to be effective in reducing perimenstrual symptoms [13]. In particular, regular exercise may help reduce depression [14], and the establishment of an exercise habit may be useful for preventing symptoms in young Japanese women who are likely to experience mental symptoms depending on menstrual cycles [15, 16].

The lack of exercise habits among young Japanese women is a serious problem [17, 18]. If the relationship between exercise habits and the severity of symptoms is clarified, non-pharmacological methods for coping with perimenstrual symptoms can be established. The objectives of this study were (1) to explore whether there is a difference in the severity of perimenstrual symptoms with and without exercise habits among Japanese young women and (2) to confirm whether exercise habit was correlated to symptoms (physical, mental, and social symptoms).

## Methods

### Study design

This observational, anonymous, cross-sectional survey was conducted from May 17, 2021 to June 14, 2021 by using Google Forms (Alphabet, Mountain View, CA, USA). The target participants of the study were young women aged 18–25 years from all regions of Japan. We recruited 10 university faculty members and 5 companies and hospitals and asked them to distribute the online survey link to organizations that had given their consent. On the first page of the questionnaire, the instructions mentioned that the question could be answered anonymously, and that a person should not answer more than once. On the same page, the purpose and methods of the survey were explained and only respondents who agreed to the survey could move on to the following page. The inclusion criteria were as follows: (1) Japanese women aged 18–25 years old, (2) resided in Japan during the survey period, and (3) agreed to participate in this research. The exclusion criteria were as follows: (1) current or previous history of gynecological or psychiatric disorders, and (2) use of daily hormone medication. This study followed the recommendations of the Checklist for Reporting Results of Internet E-Surveys [19], was conducted in compliance with the guidelines of the Declaration of Helsinki and its amendments, and was approved by the Ethical Committee for Epidemiology of Hiroshima University (E-3791).

### Survey items and data collection

The questionnaire included current and previous history of gynecological and psychiatric disorders, medication status for these disorders, current age, age at menarche, height, body weight, exercise habits, lifestyle, use of painkillers, supplementary physical activity levels, and severity of perimenstrual symptoms. Body mass index (BMI) was calculated, using weight and height (kg/m^2^).

We asked if they had an exercise habit of more than 30 min twice a week from two choices (exercise/non-exercise) [20]. Lifestyle included smoking (absence/presence), alcohol intake (low/high: consumption of < 5/≥ 5 alcoholic beverages), sleep (≥ 7 h/< 7 h), breakfast (eating/not eating), and eating between meals (eating/not eating) [21, 22]. The International Physical Activity Questionnaire–Short Form [23, 24] was used as an indicator of supplementary physical activity after the question of an exercise habit. Total physical activity was determined by asking for and calculating the average amount of walking, the weekly amount of moderate physical activity, and the vigorous physical activity (Metabolic equivalent tasks [METs]*mins/week). One Met is approximately equal to the energy consumption required for a person to sit quietly. In this study, questions regarding sedentary time were not used. The amount of physical activity was also classified into three levels: low (< 600 METs*mins/week), moderate (600–2999 METs*mins/week), and high (≥ 3000 METs*mins/week) [25].

The Menstrual Distress Questionnaire (MDQ), a standard method for measuring cyclical perimenstrual symptoms [26], consists of 46 questions and is categorized into eight subscales. We chose six subscales—pain, concentration, behavioral change, autonomic reaction, water retention, and negative affect—and excluded two subscales that were considered to be less prevalent in the Japanese women [27]. We asked about symptoms in three phases: premenstrual, during menstrual, and postmenstrual. Responses were scored using a 6-point Likert scale, which ranged from 1 (no reaction at all) to 6 (acute or partially disabling), with higher scores indicating a greater severity of perimenstrual symptoms.

### Statistical analysis

Respondents were divided into exercise and non-exercise groups, according to their exercise habits [20]. A Shapiro–Wilk test was conducted before all analyses to check for normality. Differences between the exercise and non-exercise groups in sociodemographic data and MDQ total score and subscales in each menstrual phase were detected using the Mann–Whitney U test. The chi-square test was used to compare lifestyle and physical activity levels between the two groups.

Logistic regression analysis was performed to determine the association between exercise habit and severity of perimenstrual symptoms in each of the three phases: pre-, during, and postmenstrual. The exercise group was coded as “1,” the non-exercise group as “0,” and the model was made simpler by eliminating the variables with *p* values ≥ 0.05 using a variable selection based on the probability of the Wald statistic (Wald). Odds ratios (ORs) and 95% confidence intervals (CIs) were calculated. The variance inflation coefficients were assessed and evaluated for the possibility of multicollinearity of the independent variables on multivariate regression analysis. In a previous study, it has been reported that the recommended number of respondents per variable should be ≥ 10 [28]. The sample size for the logistic regression analysis was predetermined using six independent parameters of the MDQ subscales. Therefore, a minimum of 60 respondents were needed in each group (exercise and non-exercise groups). All the Data were analyzed using IBM SPSS Statistics for Windows (version 23.0; IBM Corp., Armonk, NY, USA). The level of significance was set at *p* < 0.05.

## Results

**Fig. 1:**
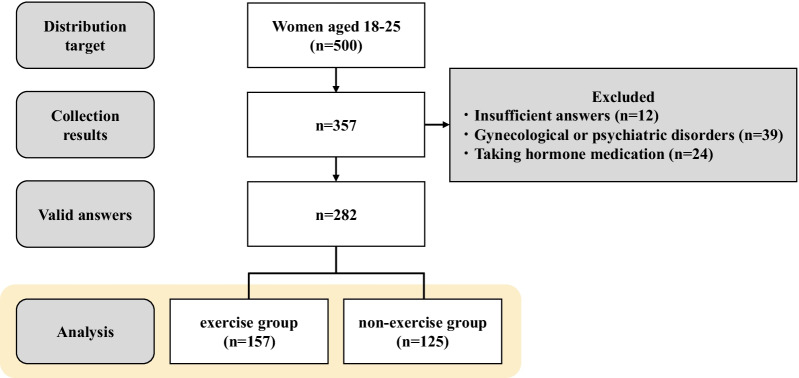
Flowchart of study participants

Of the 500 distribution targets, we received answers from a total of 357 respondents (71.4%). Of these, 12 respondents had insufficient answers, 39 respondents had a current and previous history of gynecological or psychiatric disorders, and 24 respondents were taking hormone medication. Therefore, 282 respondents (56.4%) were included in the current analysis. Respondents were divided into two groups: exercise group (n = 157) and non-exercise group (n = 125) according to their answers regarding exercise habits (Fig. 1).

### Sociodemographic characteristics, lifestyle, and physical activity levels between the exercise and non-exercise groups

The mean ages of the exercise and non-exercise groups were 21.6 years (SD = 1.9) and 21.9 years (SD = 1.6), respectively. The average age at menarche was 12.5 years (SD = 1.7) in the exercise group and 12.4 years (SD = 1.5) in the non-exercise group. BMI was 20.7 (SD = 2.1) in the exercise group and 20.1 (SD = 2.0) in the non-exercise group. There were no statistically significant differences between the two groups in terms of age, age at menarche, and BMI. In addition, there were no statistically significant differences between the groups regarding smoking, alcohol intake, sleep, breakfast, eating between meals, and using painkillers. There was a significant difference in physical activity levels between the exercise and non-exercise groups (exercise: low 28.3%, moderate 58.6%, high 33.1%; non-exercise: low 64.0%, moderate 36.0%, high 0%; *p* < 0.001) (Table 1).

**Table 1:** Sociodemographic characteristics, lifestyle and physical activity between exercise and non-exercise group

Characteristics of participants	Exercise group	Non-exercise group	χ^2^	*p* Value	Effect size
	(n = 157)	(n = 120)			
Age (years)	21.6 ± 1.9	21.9 ± 1.6		0.174	0.081
Age at menarche (years)	12.5 ± 1.7	12.4 ± 1.5		0.286	0.029
BMI (kg/m^2^)	20.7 ± 2.1	20.1 ± 2.0		0.067	0.109
*Smoking*					
Absence	147 (93.6)	120 (96.0)	0.776	0.378	0.052
Presence	10 (6.4)	5 (4.0)			
*Alcohol intake*					
Low	146 (93.0)	121 (96.8)	2.002	0.157	0.084
High	11 (7.0)	4 (3.2)			
*Sleep*					
≧ 7 h	75 (47.8)	59 (47.2)	0.009	1.000	0.006
< 7 h	82 (52.2)	66 (52.8)			
*Breakfast*					
Eating	116 (73.9)	87 (69.6)	0.634	0.426	0.047
Not eating	41 (26.1)	38 (30.4)			
*Eating between meals*					
Eating	115 (73.2)	79 (63.2)	3.273	0.070	0.108
Not eating	42 (26.8)	46 (36.8)			
*Painkillers*					
Using	75 (47.8)	58 (46.4)	0.052	0.819	0.014
Not using	82 (52.2)	67 (53.6)			
IPAQ-SF					
Low	13 (28.3)	80 (64.0)	114.233	0.001*	0.636
Moderate	92 (58.6)	45 (36.0)			
High	52 (33.1)	120 (0.0)			

Data are expressed as means ± SD, or n (%)

*BMI* body mass index, *IPAQ-SF* International Physical Activity Questionnaire Short Form

*Statistical significant

### Comparison of MDQ scores between exercise and non-exercise groups in premenstrual, during menstrual, and postmenstrual phases

**Fig. 2:**
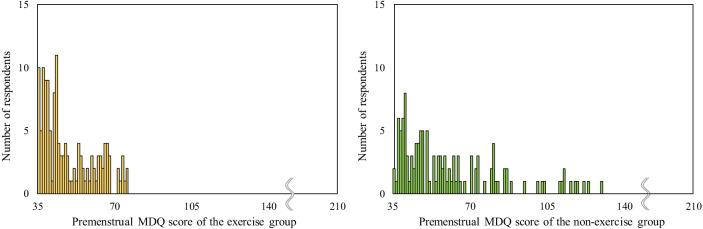
Distribution of total premenstrual Menstrual Distress Questionnaire (MDQ) scores. Left: exercise group (n = 157), Right: non-exercise group (n = 125)

**Fig. 3:**
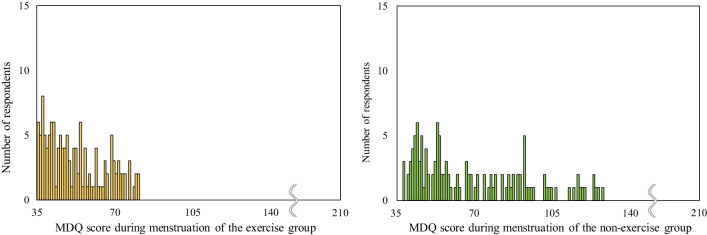
Distribution of total menstrual Menstrual Distress Questionnaire (MDQ) scores during menstruation. Left: exercise group (n = 157), Right: non-exercise group (n = 125)

The premenstrual and menstrual MDQ total scores were significantly lower and less severe in the exercise group than in the non-exercise group (premenstrual, *p* = 0.014; menstrual, *p* < 0.001) (Table 2). Focusing on the six subscales, premenstrual concentration, behavioral change, water retention, and negative affect were significantly lower in the exercise group than in the non-exercise group during the premenstrual period (*p* = 0.047, *p* = 0.040, *p* = 0.049, and *p* = 0.021, respectively). During menstruation, pain, concentration, behavioral change, water retention, and negative affect were significantly lower in the exercise group than in the non-exercise group (*p* < 0.001, *p* = 0.003, *p* < 0.001, *p* = 0.011, and *p* = 0.003, respectively). There were no significant differences in the postmenstrual MDQ total scores and all subscales. The distribution of the premenstrual MDQ total scores is shown in Fig. 2. The kurtosis and skewness were 11.737 and 3.326 in the exercise group and 6.658 and 2.497 in the non-exercise group, respectively. The MDQ total score distribution during menstruation is shown in Fig. 3. The kurtosis and skewness were 4.029 and 2.195 in the exercise group and 4.920 and 2.189 in the non-exercise group, respectively.

**Table 2:** Comparison of MDQ scores between exercise and non-exercise groups

	Premenstrual				During menstruation				Postmenstrual			
	Exercise group	Non-exercise group	*p* Value	Effect size	Exercise group	Non-exercise group	*p* Value	Effect size	Exercise group	Non-exercise group	*p* Value	Effect size
Total	54.1 ± 19.1	60.2 ± 23.3	0.014*	0.146	57.5 ± 19.3	68.4 ± 25.0	< 0.001*	0.231	41.7 ± 14.5	43.2 ± 16.0	0.358	0.055
Pain	10.0 ± 4.5	10.6 ± 4.8	0.237*	0.070	12.2 ± 5.3	14.6 ± 6.0	< 0.001*	0.217	7.9 ± 4.0	8.0 ± 3.8	0.721	0.021
Concentration	10.0 ± 3.7	11.2 ± 5.0	0.047*	0.118	10.5 ± 3.6	12.3 ± 5.2	< 0.003*	0.176	8.7 ± 2.2	9.2 ± 3.0	0.467	0.043
Behavioral change	9.1 ± 4.2	10.3 ± 5.2	0.040*	0.122	10.0 ± 4.6	12.5 ± 5.6	< 0.001*	0.236	6.1 ± 2.6	6.7 ± 3.6	0.266	0.066
Autonomic reaction	4.6 ± 1.6	4.7 ± 1.6	0.380*	0.052	5.1 ± 2.0	5.6 ± 2.5	< 0.068*	0.109	4.4 ± 1.7	4.4 ± 1.3	0.095	0.099
Water retention	8.2 ± 3.7	9.0 ± 3.7	0.049*	0.117	7.6 ± 3.4	8.7 ± 3.7	< 0.011*	0.152	5.3 ± 2.6	5.6 ± 2.4	0.138	0.088
Negative affect	12.3 ± 5.9	14.5 ± 7.6	0.021*	0.137	12.0 ± 4.8	14.7 ± 7.3	< 0.003*	0.179	9.2 ± 3.2	9.4 ± 3.7	0.911	0.007

Data are expressed as means ± SD

*MDQ* Menstrual Distress Questionnaire

*Statistical significant

### Multiple logistic analysis for premenstrual and during menstrual symptoms

To assess the relationship between exercise habits and MDQ subscales, multiple logistic analysis was performed with the exercise and non-exercise groups as variables in the premenstrual and during menstrual groups. The possibility of multicollinearity of independent variables in multivariate regression analysis was evaluated by calculating the variance inflation factor, and no independent variables were excluded. In the analysis of the premenstrual period, statistically significant associations with exercise habits were detected in negative affect (β = -0.048, *p* = 0.018, OR = 0.953, 95% CI 0.919–0.988). There was no statistically significant association between concentration (*p* = 0.800), behavioral change (*p* = 0.997), and water retention (*p* = 0.678) (Table 3). On analysis of the period during menstruation, a statistically significant association with exercise habit was detected in behavioral change (β = − 0.096, *p* < 0.001, OR = 0.909, 95% CI 0.866–0.953). No significant relationship was found in the other items of pain, concentration, water retention, and negative affect (Table 4).

**Table 3:** Multiple logistic analysis for association of an exercise habit with premenstrual MDQ subscales

Variables	β	SE	Wald	df	*p* Value	Odds ratio	95% CI	
							Lower	Upper
Concentration	− 0.012	0.047	0.064	1	0.800	0.988	0.902	1.083
Behavioral change	− 0.003	0.050	0.003	1	0.956	0.997	0.905	1.099
Water retention	− 0.016	0.040	0.173	1	0.678	0.984	0.910	1.063
Negative affect	− 0.048	0.018	10.125	1	0.009*	0.953	0.919	0.988

Variation inflation factor: concentration, 2.623; behavioral Change, 3.526; water retention, 1.451; negative affect, 2.920

*MDQ* Menstrual Distress Questionnaire, *β* partial regression coefficient, *SE* standard error, *df* degree of freedom

*Statistical significant

**Table 4:** Multiple logistic analysis for association of an exercise habit with MDQ subscales during menstruation

Variables	β	SE	Wald	df	*p* Value	Odds ratio	95% CI	
							Lower	Upper
Pain	− 0.027	0.033	0.669	1	0.413	0.973	0.912	1.039
Concentration	− 0.013	0.045	0.083	1	0.773	0.987	0.904	1.078
Behavioral change	− 0.096	0.024	15.345	1	< 0.001*	0.909	0.866	0.953
Water retention	0.005	0.045	0.013	1	0.909	1.005	0.920	1.098
Negative affect	− 0.013	0.036	0.129	1	0.719	0.987	0.919	1.060

Variation inflation factor: pain, 2.270; concentration, 2.490; behavioral change, 2.920; water retention, 1.660; negative affect, 3.042

*MDQ* Menstrual Distress Questionnaire, *β* partial regression coefficient. *SE* standard error, *df* degree of freedom

*Statistical significant

## Discussion

This is the first study to show the relationship between the exercise habit and perimenstrual symptoms in young Japanese women. The main findings were as follows: (1) The total MDQ score of the exercise group was lower than that of the non-exercise group in the premenstrual and menstrual periods and (2) the “negative affect” before menstruation and the “behavior change” during menstruation were associated with an exercise habit and showed less severity.

Premenstrual and menstrual symptoms were lower in the exercise group than in the non-exercise group. It is interesting to note that having an exercise habit may reduce various symptoms related to menstruation, including physical, mental, and social symptoms. A previous study has shown that many women experience a variety of perimenstrual symptoms and not just pain [1]. While exercise has been known to reduce chronic pain, swelling, and depression [11, 12, 14], the relationship between symptoms associated with the menstrual cycle and exercise habits is unclear. This study confirmed that having an exercise habit may also be effective in reducing symptoms caused by the menstrual cycle. Many women suffer from symptoms that are not severe enough to require a hospital visit [5, 29], and the finding that an exercise habit may reduce various perimenstrual symptoms is very useful for young women.

MDQ subscales that were related to an exercise habit were “negative affect” and “behavioral change before and during menstruation.” The "negative affect" comprises mental symptoms, such as depression and irritability [24], and the "behavioral change" includes symptoms such as avoiding interaction with others [24]. One reason for the reduction in “negative affect” and “behavioral change” may be related to the secretion of estradiol in the body as a result of exercise [30, 31]. Estradiol is a female hormone that is thought to be more potent than other estrogens. Estradiol was reported to increase serotonin secretion in the brain [32] and to reduce negative emotions [33]. Low-intensity exercise, such as running at a pace that is comfortable for oneself, increases estradiol secretion [32, 33]. Hence, in this study, the criteria of exercise habit might have also stimulated estradiol secretion. Additionally, chronic increases in cortisol levels are a known cause of mental and social stress, and exercise is effective for the control of cortisol secretion [34]. A previous study showed that 30 min of exercise will moderate cortisol increases during the occurrence of subsequent psychosocial stressors [35]. Thus, it is possible that the exercise habit reduced cortisol reactivity and alleviated the effects of the psychosocial aspect of the stressor of menstruation. In addition, a previous study showed that people with an exercise habit have a higher level of daily well-being [36]. It was inferred that the subjects of this study also had an opportunity to create a positive mood and were freed from negative thinking processes by having an exercise habit. Having an exercise habit may also promote social interaction and create opportunities to go outside, such as going for a walk and engaging in activities with friends. It has been suggested that it is difficult to exercise alone at home without high motivation and exercise equipment [37]. A previous study conducted in China showed that encouragement from those around one is effective to continue exercising [38]. The interaction is that exercising encourages people to connect with others, and connections with others help establish exercise habits. Therefore, internal and external factors may have influenced the reduction in mental and social symptoms in the exercise group.

The results of this study, which indicate that the severity of perimenstrual symptoms differs between women with and without an exercise habit, suggest that establishing an exercise habit may be an effective non-pharmacological coping strategy. In the past, many exercise standards recommended for health promotion were of high intensity, but in recent years, the intensity has tended to decline because even low-intensity exercise has been shown improve health [31, 39]. Many Japanese women avoid exercising due to their busy work and study schedules [40], and even young women are at risk for lifestyle-related diseases and locomotive syndrome due to a lack of exercise [17, 18]. Therefore, the absence of exercise habits among young women is a serious problem. The standard of 30 min or more twice a week, which was used as the criteria in this study, is considered to be a relatively easy way for those who do not have an exercise habit to start a new exercise program. This study provides useful information for young women who want to prevent perimenstrual symptoms but do not have an exercise habit, and it may encourage young women to start exercising. In addition, it has been pointed out that perimenstrual symptoms of young women in Japan are characterized by a tendency to exhibit "negative affect" [16], and that there is a need for further education on coping strategies for psychological symptoms associated with menstruation. This study, which showed that the presence or absence of an exercise habit had a significant effect on the "negative affect" and "behavioral change" of the MDQ score, may be used to educate young people on how to cope with psychological and social symptoms.

This study has some limitations. First, it did not consider the effects of excessive exercise on menstrual and perimenstrual symptoms. Second, we asked respondents to complete all the questionnaires regardless of their current stage of the menstrual cycle, so we could not analyze the data in each menstrual phase because it was a cross-sectional survey. Third, the influence of recall bias cannot be excluded because this was a retrospective survey. We have confirmed that there is no significant bias in the proportions of premenstrual, menstrual, and postmenstrual in each group. Then, it was possible that questionnaire bias may have occurred due to the length of the questionnaire [41]; however, the questions were clearly expressed in plain words. The longitudinal effects of exercise habit on perimenstrual symptoms were also unknown due to the cross-sectional nature of the study. Finally, there was a selection bias because the survey was conducted online among Japanese women. In the future, we will examine in more detail the duration, frequency, intensity, and type of exercise that is effective in reducing perimenstrual symptoms.

## Conclusion

Our study investigated the relationship between the severity of perimenstrual symptoms and regular exercise habits in young Japanese women. The premenstrual and menstrual MDQ total scores were significantly lower and less severe in the exercise group than in the non-exercise group. A multiple logistic analysis showed that the “negative affect” before menstruation and “behavior change” during menstruation of MDQ subscales were associated with an exercise habit, which means that there are few fluctuations in mood and behavioral changes, such as avoiding interaction with others, depending on the menstrual cycle. Most other premenstrual and during menstrual MDQ subscales were significantly lower in the exercise group than in the non-exercise group; however, these had no significant relationship with exercise habit in the multiple logistic analysis. Based on these findings, our results suggest that exercise habits may reduce the severity of perimenstrual symptoms. This study’s findings may encourage health promotion for young women and the establishment of non-pharmacological methods of coping with perimenstrual symptoms.

## Supplementary Information

**Additional file 1.** The raw data used in the analysis of this study.

## Data Availability

All data generated or analysed during this study are included in this published article [and its additional files].

## Abbreviations

BMI: Body mass index
CI: Confidence interval
MDQ: Menstrual Distress Questionnaire
OR: Odds ratio
SD: Standard deviation
